# Aggressive fibromatosis of anterior maxilla

**DOI:** 10.4103/0973-029X.80019

**Published:** 2011

**Authors:** Devi C Shetty, Aadithya B Urs, Puneet Ahuja, Seema Sikka

**Affiliations:** *Department of Oral and Maxillofacial Pathology, I.T.S.-C.D.S.R., Muradnagar, Ghaziabad, India*; 1 *I.T.S Dental College, Greater Noida, U.P., India*

**Keywords:** Aggressive, fibromatosis, maxilla

## Abstract

Aggressive fibromatosis is a comparitively rare tumor with unpredictable growth and varying local recurrence rates. It does not develop distant metastases but locally it shows an aggressive and infiltrative behavior. Clinically, aggressive fibromatosis manifests as a painless, firm, often rapidly enlarging mass, fixed to underlying bone or soft tissue. It is never encapsulated. Histologically, it is rich in collagen and fibroblastic cells that are devoid of hyperchromatic or atypical nuclei, but with more variable cellularity in different tumor sections.

## INTRODUCTION

The term fibromatosis refers to a group of fibrous tumors or tumor like lesions of soft tissue that share similar microscopic characteristics and possess an intermediate biologic potential between benign and malignant lesions.[1] Fibromatosis of head and neck makes up roughly 10-12% of reported cases of extra-abdominal fibromatosis; the supraclavicular area is the most commonly affected site.[2] The oral structures *per se* are not often the site of origin. Fibromatoses are nonmetastasizing but may exhibit both rapid growth and visceral involvement. Spontaneous regression has been described but rare tumors mimic a malignancy in their tendency to occur locally.[3]

Pathologically, fibromatosis has a deceptively bland appearance. It is however associated with an infiltrative growth pattern that results in difficulty in complete excision and propensity for recurrence.[4]

Clinical differentiation of desmoids from malignant tumors may be difficult in view of the local invasive growth of the former. There is a positive correlation between clear histologic margins and long-term disease-free survival only in relapse cases.[5]

Here, we report a case of fibromatosis involving the anterior maxilla. The pathological diversity in all the areas was analyzed in great detail with emphasis on the histopathologic parameters which aid in its diagnosis.

## CASE REPORT

A 21-year-old male reported with a chief complaint of swelling and pain on left side of the face since 1 month. Past history revealed that the pain subsided on intake of medicines prescribed by a local practitioner, but the swelling did not subside. Patient had a history of pus discharge since 2 days. Intraoral examination revealed swelling of size 3 × 3 cm in the left anterior maxillary region, with ill-defined borders. On palpation, it was soft in consistency. No lymph node involvement was observed. On radiological examination, an osteolytic lesion with irregular margins was seen in the upper occlusal radiograph. The computed tomography (CT) scan revealed soft tissue mass perforating the anterior wall of maxilla and palate. The provisional diagnosis was of central giant cell granuloma.

**Figure 1:**
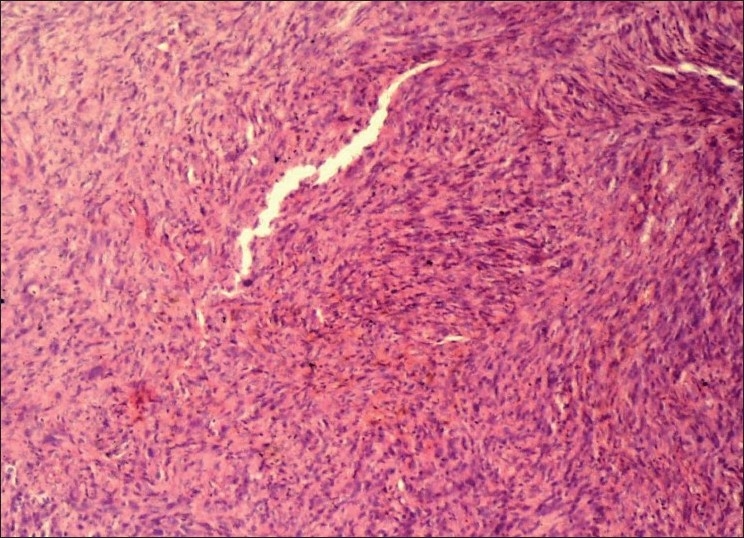
Photomicrograph showing fasciculated arrangement of cells (H and E, 10×)

**Figure 2:**
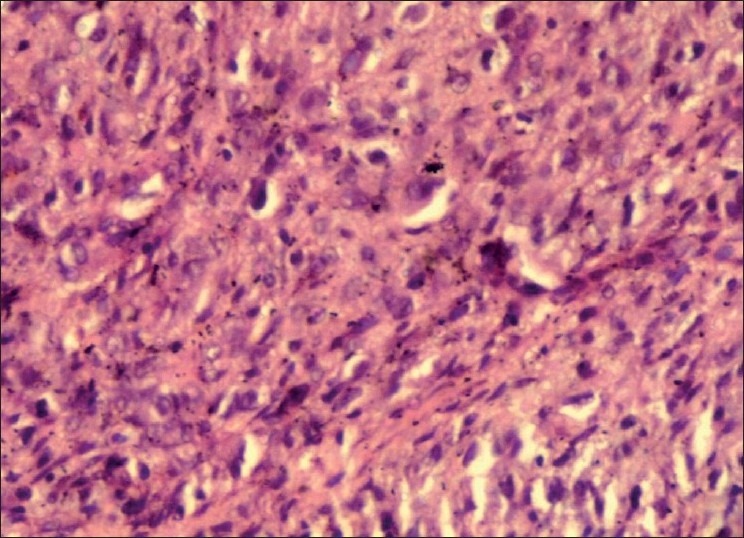
Photomicrograph showing cellular and nuclear pleomorphism (H and E, 40×)

**Figure 3:**
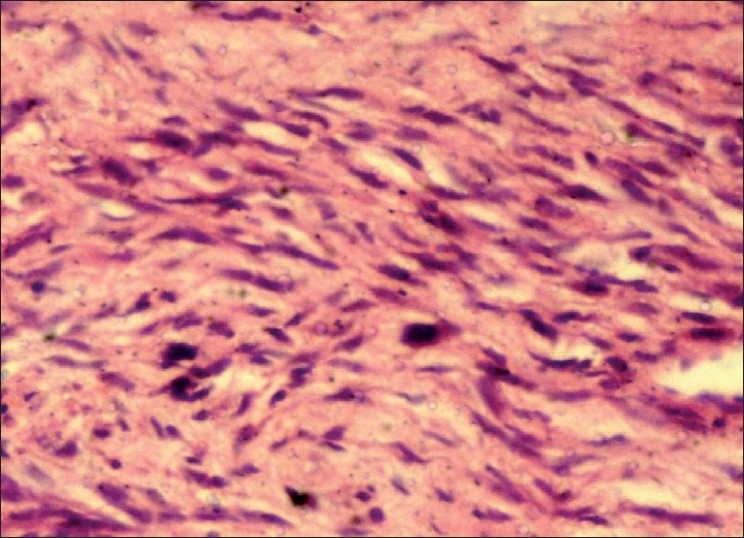
Photomicrograph showing bland spindle-shaped cells (H and E, 40×)

**Figure 4:**
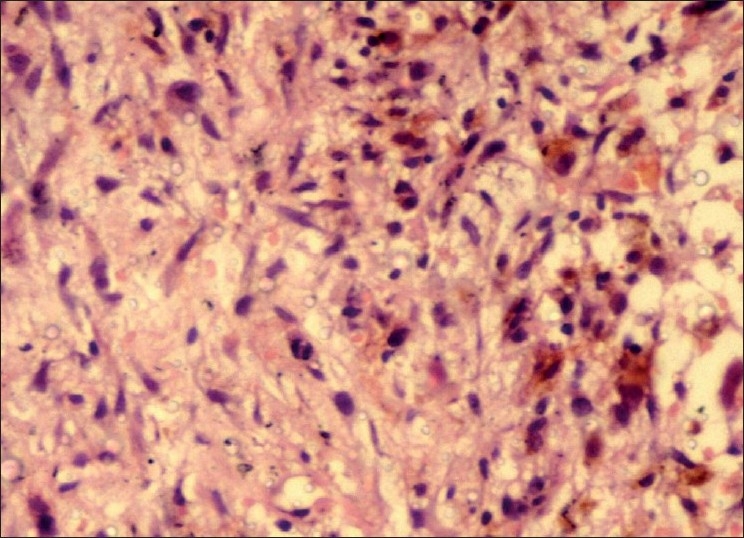
Photomicrograph showing two typical mitotic figures visible in the center (H and E, 40×)

An incisional biopsy was performed and gross examination showed four brownish soft tissue bits with one larger bit measuring 3 × 1.5 cm and three approximately equal bits measuring 1 × 1 cm. Microscopic examination revealed a connective tissue proliferation of predominantly fibroblastic cells in a background of moderate amount of collagen tissue. The pattern of arrangement varied from sheets to storiform, and to a certain extent, was in a fascicular pattern [Figure 1]. The cells were large, oval and fibroblastic in nature, but pleomorphism was abundant. Pleomorphic cells [Figure 2] showed a range from elongated flattened nuclei to a large giant cell like elongated nuclei. Cells showed moderate to abundant cytoplasm. A few areas of the section were predominantly spindle and wavy in nature [Figure 3]. The blood vessels seemed to be constricted due to the high proliferative nature of the cells around. Nucleus of the tumor cells showed a clear nuclear membrane, but in some areas, condensation of the nuclear material and two or more nucleoli were seen. Mitotic figures were present but were not seen uniformly throughout the section [Figure 4]. A final diagnosis of aggressive fibromatosis was assigned.

## DISCUSSION

The fibromatosis constitutes part of a spectrum of poorly understood proliferative lesions whose histologic features overlap to such an extent that the pathologist may be more influenced by the anatomic location of the lesion, sex and clinical behavior than by the histologic appearance in rendering his or her diagnosis. Wherever it occurs, the diagnosis and management of fibromatosis are always sources of concern.[6]

It has been defined as a non-neoplastic spindle cell proliferation of childhood, which may be locally aggressive but has no metastatic potential. The natural history is of initial rapid growth and local aggression.[3] Enzinger and Weiss divided the fibromatosis into two broad categories: superficial and deep. The fibromatoses that occur in the head and neck including those that involve oral and paraoral structures are considered under the heading of extra-abdominal fibromatosis. Infantile fibromatosis is the childhood counterpart of extra-abdominal fibromatosis.[1]

The histopathologic differentiation between aggressive fibromatosis and other closely related spindle cell lesions like fibrosarcoma, neurofibroma, nodular fascitis, fibrous histiocytoma and infantile myofibromatosis are a challenge to the pathologists as it requires expertise to differentiate the finer details. But the major challenge in dealing with the lesions of fibromatosis is to avoid an overdiagnosis of fibrosarcoma and an underdiagnosis of reactive fibrosis. Fibromatosis has a more uniform growth pattern, more mature cells and a paucity of mitosis compared with fibrosarcoma. Reactive fibrosis such as that following injury or trauma has a more variable growth pattern than fibromatosis and may show areas of focal hemorrhage or hemosiderin deposition.[1]

The grade I fibrosarcoma is usually discernable from fibromatosis by the presence of occasional larger nuclei with ominous chromatin clumping, greater cellularity, greater mitotic activity and thin rather than thick collagen bundles.[6] Immunohistochemistry is of little help in differential diagnosis because positive immunostaining against vimentin can be observed in all fibrous connective tissue tumors.[7] Mitotic figures are rare and the finding of more than one mitotic figure per high power field or atypical mitotic figures should raise the suspicion of fibrosarcoma. Since on rare occasions, features of fibromatosis and fibrosarcoma are found together in the same neoplasm, careful sampling of the tumor is mandatory for a reliable diagnosis. Clinical considerations are of little help in distinction of fibromatosis and fibrosarcoma because both tumors may occur at the same location and in the same age group. Also, it is notoriously difficult to separate fibromatosis from well-differentiated fibrosarcoma, especially in infants and juveniles when fibromatosis is characterized by higher mitotic rates than in adults. Indeed, doubts have been expressed as to whether this distinction can be made at all.[6]

The storiform–pleomorphic variant of malignant fibrous histiocytoma also shows frequent transitions from storiform to pleomorphic pattern. In its classic form, a lesion of malignant fibrous histiocytoma consists of plump spindle cells arranged in short fascicles in a cartwheel or storiform pattern around slit-like vessels. These differ from other similar lesions by the presence of occasional plump histiocytic cells, numerous typical and atypical mitotic figures and secondary elements including xanthoma cells and modest number of chronic inflammatory cells. Another characteristic feature is the presence of large number of giant cells with multiple hyperchromatic irregular nuclei.[8]

Authors have propounded that since soft tissue and intraosseous lesions are histologically indistinguishable and since in the maxilla and mandible, origin in bone or soft tissue is uncertain, the term desmoplastic should not be used in the area of head and neck but all the lesions should be termed desmoid fibromatosis.[6]

A small panel of antibodies including S-100 protein, smooth muscle actin (SMA), desmin and vimentin would in most cases help in establishing the diagnosis. Fibromatosis is generally positive for vimentin. However, it should be pointed out that immunohistochemical studies have shown myofibroblastic differentiation in some cases of fibromatosis. Thus, in such cases, SMA would also be positive along with vimentin. A case of fibrous histiocytoma is positive for vimentin and can be variably positive for actin.[1] Histopathologically, our differential diagnosis narrowed down to aggressive fibromatosis and low-grade fibrosarcoma. So, in our case, immunohistochemistry was not utilized since vimentin is positive for both the lesions.

The final diagnosis of aggressive fibromatosis was based on a number of factors which included spindle-shaped monotonous population of fibroblasts arranged in a whorl like and fasciculated pattern and the presence of collagen. The cells were mature in appearance and the presence of few typical mitotic figures was noticed.

## References

[1] Fowler CB, Hartman KS, Brannon RB (1994). Fibromatosis of the oral and paraoral region. Oral Surg Oral Med Oral Pathol.

[2] Masson JK, Soule EH (1966). Desmoid tumours of the head and neck. Am J Surg.

[3] Baerg J, Murphy JJ, Magee JF (1999). Fibromatosis: Clinical and pathological features suggestive of recurrence. J Pediatr Surg.

[4] Tse GM, Chan KF, Ahuja AT, King AD, Pang PCW, To EWH (2001). Fibromatosis of the head and neck region. Otolaryngol Head Neck Surg.

[5] Watzinger F, Turhani D, Wutzl A, Fock N, Sinko K, Sulzbacher I (2005). Aggressive fibromatosis of the mandible: A case report. Int. J. Oral Maxillofac Surg.

[6] Vally IM, Altini M (1990). Fibromatosis of the oral and paraoral soft tissue and jaws- Review of literature and report of 12 new cases. Oral Surg Oral Med Oral Pathol.

[7] Seper L, Burger H, Vormoor J, Joos U, Kleinheinz J (2005). Aggressive fibromatosis involving the mandible - Case Report and review of literature. Oral Surg Oral Med Oral Pathol Oral Radiol Endod.

[8] Enzinger FM, Weiss SW (1988). Malignant fibrous Histiocytic Tumors. Soft tissue tumors.

